# The Role of Src Kinase in Macrophage-Mediated Inflammatory Responses

**DOI:** 10.1155/2012/512926

**Published:** 2012-11-11

**Authors:** Se Eun Byeon, Young-Su Yi, Jueun Oh, Byong Chul Yoo, Sungyoul Hong, Jae Youl Cho

**Affiliations:** ^1^Department of Genetic Engineering, Sungkyunkwan University, Suwon 446-746, Republic of Korea; ^2^Research Institute and Hospital, National Cancer Center, Goyang 410-769, Republic of Korea

## Abstract

Src kinase (Src) is a tyrosine protein kinase that regulates cellular metabolism, survival, and proliferation. Many studies have shown that Src plays multiple roles in macrophage-mediated innate immunity, such as phagocytosis, the production of inflammatory cytokines/mediators, and the induction of cellular migration, which strongly implies that Src plays a pivotal role in the functional activation of macrophages. Macrophages are involved in a variety of immune responses and in inflammatory diseases including rheumatoid arthritis, atherosclerosis, diabetes, obesity, cancer, and osteoporosis. Previous studies have suggested roles for Src in macrophage-mediated inflammatory responses; however, recently, new functions for Src have been reported, implying that Src functions in macrophage-mediated inflammatory responses that have not been described. In this paper, we discuss recent studies regarding a number of these newly defined functions of Src in macrophage-mediated inflammatory responses. Moreover, we discuss the feasibility of Src as a target for the development of new pharmaceutical drugs to treat macrophage-mediated inflammatory diseases. We provide insights into recent reports regarding new functions for Src that are related to macrophage-related inflammatory responses and the development of novel Src inhibitors with strong immunosuppressive and anti-inflammatory properties, which could be applied to various macrophage-mediated inflammatory diseases.

## 1. Introduction

Inflammation is a complex biological response to various harmful stimuli and is accompanied by various symptoms including redness, swelling, heat, and pain. A major cause of inflammation stems from the infection of local tissue with pathogens, such as bacteria, viruses, and fungi; inflammation is the process that attracts various immune cells to the injured tissues and removes the infecting pathogens. These processes involve closely related chemical mediators, such as nitric oxide (NO), reactive oxygen species (ROS), prostaglandin E2 (PGE_2_), histamine, and cytokines including TNF-*α* and various interleukins [1]. There are two types of inflammatory responses acute inflammation and chronic inflammation. Acute inflammation is a rapid and temporary host response induced by leukocytes and plasma proteins containing antibodies in the infected or injured tissues. Chronic inflammation is persistent inflammation characterized by tissue injury and attack and has a longer recovery time. The chronic response may increase damage to the tissues and organs, resulting in the onset of diseases, such as rheumatoid arthritis, tuberculosis, arteriosclerosis, and pulmonary fibrosis.

Macrophages are generated by the differentiation of monocytes and are located in several tissues. Depending on their location, macrophages have different names, for example, Kupffer cells in the liver, alveolar macrophages in the lung, microglia in the central nerve system (CNS), and osteoclasts in the bone. The cells are activated by various stimuli through receptors, such as Toll-like receptors and receptors that recognize the antigenic ligands from microorganism, the cytokines secreted by immune cells, and other chemical mediators. Activated macrophages induce the production of lysosomal enzymes, NO, ROS, cytokines, growth factors, and other inflammatory mediators.

Src kinase (Src) is a protooncogene encoding a protein tyrosine kinase. Originally, Bishop and Varmus discovered Src in 1967. The Src gene (*src*) is similar to the *v-src* from the Rous sarcoma virus. Src phosphorylates a tyrosine residue on its target protein, and Src activity is regulated by the autophosphorylation of its own tyrosine residues. Src is classified as a nonreceptor tyrosine kinase belonging to the Src family kinases, which include nine members exhibiting similar functions and structures. Src family kinases play critical roles in the progression of cancers; however, recent studies have reported that Src is also involved in the inflammation-related signaling pathway.

In this paper, we provide a general introduction to the roles of Src as an oncoprotein, focusing on the in-depth investigation of the role of Src in macrophage-mediated inflammatory diseases. Furthermore, we provide a perspective on the feasibility of using plant extracts and other natural products as therapeutic drug candidates for the treatment of inflammatory diseases.

## 2. Src 

### 2.1. Src Family Kinases

The Src family members are classified as non-receptor tyrosine kinases consisting of 9 members (Table 1). Src, Fyn, Yes, and Frk exhibit ubiquitous expression, whereas Blk, Fgr, Hck, Lck, and Lyn are expressed only in restricted cells [2]. Several Src family members (Blk, Fgr, Fyn, Hck, Lck, Lyn, and Yes) are important in the signaling pathways in cells of hematopoietic lineages [3, 4]. For example, Lck and Fyn, which are expressed in T cells, are the first signaling molecules activated downstream of the T-cell receptor. In mature monocytes and macrophages, inflammatory stimuli including lipopolysaccharide (LPS) induce the expression of Hck, Lyn, and Fgr [4, 5]. 

### 2.2. Src Structure and Regulation of Src Activity

**Figure 1:**
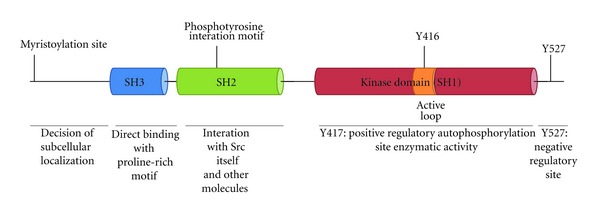
The structure of Src.

Src family kinases exhibit a similar structure comprised of (i) an N-terminal region containing myristoylation and palmitoylation sites that determine the cellular localization and confer unique functions to the Src family members [4, 6, 7]; (ii) a Src homology 3 (SH3) domain that binds directly to proline-rich regions; (iii) a Src homology 2 (SH2) domain that interacts with a phosphotyrosine residue on either itself or other proteins; (iv) a linker domain connecting the SH2 and kinase domains, which interacts with the SH3 domain [4, 7, 8]; (v) a kinase domain responsible for the enzymatic activities, containing an activation loop (A-loop) that has an autophosphorylation site at Tyr-416 and regulates the association with substrates; (vi) a C-terminal tail containing the negative-regulatory site (Tyr-527) that binds to the SH2 domain (Figure 1) [4, 7, 9]. 

**Figure 2:**
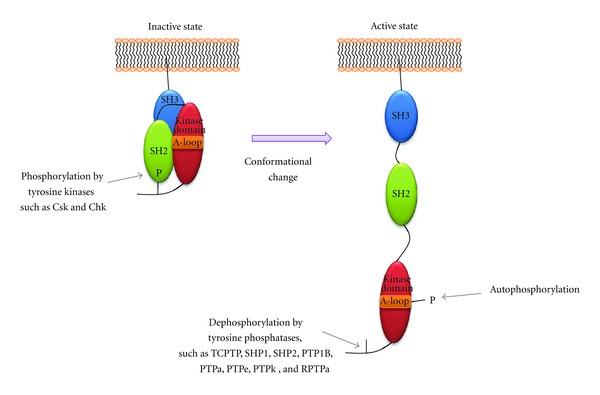
Schemata of conformational changes of Src.

Src activity is regulated by the structural changes caused by the phosphorylation and dephosphorylation of its tyrosine residues (Figure 2). Through the interaction of the SH2 or SH3 domain with the other domains, Src changes its structure and exhibits different levels of activity. The inactive structure of Src occurs when the phosphorylated Tyr-527 residue in the C-terminal tail binds to the SH2 domain and simultaneously, the SH3 domain binds to the polyproline motifs of the linker domain. In this conformation, the activation loop adopts a compact structure that fills the catalytic site, thereby precluding the binding of ATP and substrates, covering Tyr-416, and prohibiting the activation of autophosphorylation. Conversely, when Tyr-416 is autophosphorylated, Src adopts an active structure by releasing the interaction of the SH2 or SH3 domain with the other domains and displacing the p-Tyr-416 from the binding site of SH2, allowing the substrate or associating proteins (Table 2) to be accessed. Therefore, the phosphorylation of the tyrosine residues in the Src family kinases is the critical process that regulates enzymatic activity [7, 10–12].

Src activity can also be regulated by other tyrosine kinases. The C-terminal Src kinase (Csk) and the Csk homology kinase (Chk) are 2 main tyrosine kinases responsible for the phosphorylation of the inhibitory Tyr-527 in Src [13, 14], and Chk forms a complex with the autophosphorylated form of Src by noncovalent binding. Consequently, Chk blocks the kinase activity of Src [15]. Although little is known about the regulation of Src activity by protein tyrosine phosphatases (PTPs), a number of PTPs including the T-cell protein tyrosine phosphatase (TCPTP), the SH2 domain-containing protein tyrosine phosphatases 1 and 2 (SHP1 and SHP2), PTP1B, PTP*α*, PTP*ε*, PTP*κ*, and receptor-PTP*α* (R-PTP*α*) are involved in the regulation of Src activity through the dephosphorylation of the C-terminal tyrosine in Src [7, 16–19]. Therefore, it is possible that Src activity is regulated not only by itself through autophosphorylation but also by other tyrosine kinases, such as PTPs. 

### 2.3. Functions of Src in Inflammatory Cells

**Figure 3:**
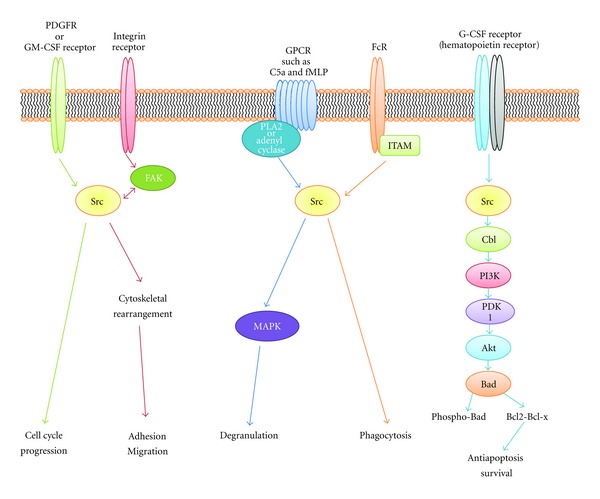
The role of Src in immune cells. A combination of antigens, cytokines, adhesion molecules, lipid factors, and their different receptors relevant to immune cell development and inflammatory responses. Regardless of the stimulus and the receptor type, Src plays a critical role in recruiting a number of cell signaling molecules. Initiated and activated by the receptor, Src increases and varies the signal. Depending on the signal received, multiple pathways that influence cell migration, adhesion, phagocytosis, cell cycle, and cell survival are activated.

Several studies have reported that Src is involved in a variety of immunologic processes (Figure 3), such as immune cell development, proliferation, adhesion, migration, chemotaxis, phagocytosis, and survival [20]. In myeloid cell development, although the role of Src is not clear, Src enhances cell cycle progression by accelerating the secretion of growth factors. Several studies have indicated that Src increases cell proliferation through the activation of receptor tyrosine kinases (RTKs), such as the platelet-derived growth factor receptor or macrophage colony-stimulating factor receptor [21–23]. Among the cells of the immune system, monocytes and macrophages are unique in that they move via multiple steps, by adhesion to the vascular endothelium, migration through the vascular membrane, and recruitment at the infected site. Adhesion, migration, and recruitment are mediated by reciprocal interactions between many types of adhesion molecules on immune cells, such as selectins, gangliosides, integrins, and other adhesion molecules [24]. In addition to adhesion, integrin-FAK-Src signaling pathways can promote various changes in cell morphology, cell cycle progression, and gene transcription [25–27]. Immune cells such as neutrophils recognize chemotactic gradients via GPCRs, the receptors for C5a or fMLP. Although the essential function of the GPCR is to activate G proteins and effector enzymes involving phospholipase A2 or adenyl cyclase, crosstalk with other proteins, including Src, has also been reported [28]. For example, fMLP-induced degranulation is decreased significantly in neutrophils treated with the Src inhibitor PP1 and in Src-deficient cell lines [29]. Src may participate in MAP kinase signaling through the *βγ* subunits of the GPCR. Gutkind reported crosstalk between the GPCR and Src in PI3-kinase *γ* signaling that is activated by the *βγ* subunits of GPCR [21, 30]. Src, through its involvement with phagocytosis, cell cytotoxicity, and the secretion of inflammatory mediators, is also responsible for host defense mechanisms. Macrophages are major players in both phagocytosis and antibody-dependent cell-mediated cytotoxicity (ADCC) [21, 31–33]. After phagocytes migrate to and infiltrate the infection sites, they engulf bacteria, fungi, or viruses. Phagocytosis occurs through the binding of Fc receptors to immunoglobulins. Specifically, Fc*γ* receptor cross-linking is induced by the phosphorylation of the immunoreceptor tyrosine-based activation motifs (ITAMs) located in the cytoplasmic tail of tyrosine kinase receptors. Src, Fyn, Fgr, Lck, and Lyn are expressed in phagocytes, where they form complexes with inactivated Fc*γ*Rs. Src-deficient cells are less effective than wild-type cells at mediating phagocytosis [21, 34–37]. Hematopoietins, such as G-CSF, erythropoietin, or IL-3, diminish apoptosis in blood cell progenitors and are critical for survival [21, 38]. The expression of *v-Src* plays a critical role in IL-3-mediated survival and proliferation [21, 39]. As with T-cell receptor (TCR) proteins, integrins, Fc receptors (FcR), and G-CSF receptors lead to dimerization and rapid changes in tyrosine autophosphorylation as well as to the synthesis of a variety of signaling proteins. Korade-Mirnics and Corey reported that apoptosis is blocked mainly by a signaling pathway consisting of Src-Cbl-PI3-kinase-PI3-kinase-dependent kinase (PDK)-Akt-Bad; this pathway is enhanced by various growth factor responses [21]. In Src-dependent signaling, Akt activation is downregulated by the carboxyl-terminal region of the G-CSF receptor. Src may also induce apoptosis. Therefore, Src either induces or inhibits apoptosis, depending on the type of stimuli [21, 40]. In the inflammatory response, Src influences a broad range of immune cell activities through the interaction with various receptors and ligands in inflammation. 

## 3. The Role of Src in the Inflammatory Signaling Pathway

### 3.1. TLR-Mediated Signaling Pathway

Cells recognize both invading pathogens and injured tissue via members of the pattern recognition receptor family (PRRs). In mammalian systems, PRRs consist of 3 major families of molecules, Toll-like receptors (TLRs), the nucleotide oligomerization domain (NOD) family, and the caspase recruitment domain (CARD) family. The first role of NODs and CARDs appears to be as intracellular molecules that recognize intracellular bacteria and viruses and tissue damage; however, research regarding the effect of diminished signaling by these molecules is in its infancy. In contrast, TLRs are expressed both intracellularly and on the cell surface, and recently, significant studies regarding the molecular mechanisms of TLRs have been published [41, 42].

The TLR family is comprised of at least 10 members (Table 3). The molecules specifically recognize a range of bacterial, viral, fungal, and endogenous ligands and therefore, promote responses against a wide range of physical and environmental injuries [43]. TLRs are transmembrane molecules, and their molecular mechanism is mediated by the association of their internal domains with various complexes of the following 5 different adaptor molecules: myeloid differentiation primary response gene 88 (MyD88), MyD88 adaptor-like/TIR domain-containing adaptor protein (MAL), Toll-IL-1-resistance domain-containing adaptor inducing interferon *β* (TRIF), TRIF-related adaptor molecule (TRAM), and sterile-*α* and armadillo motif-containing protein (SARM) [44]. To date, TLR4 signaling appears to be the most complex and uses all 5 adaptors [45]. MAL and MyD88 pair to enhance the activation of IRAK1, which in turn activates Traf6 and IRAK4 leading to the activation of nuclear factor (NF)-*κ*B. The activation of the TRAM/TRIF pathway is associated with the phosphorylation/activation of IRF3, resulting in the induction of type 1 interferon and an antiviral pathway. In contrast, SARM plays the role of a negative regulator of TLR signaling. SARM forms a complex with TRIF directly and negatively regulates the function of TRIF by blocking TRIF-mediated IRF7 and NF-*κ*B activities, resulting in the inhibition of inflammatory gene activation. Therefore, the activation of these adaptor molecules initiates a signaling cascade that leads to changes in gene expression in stimulated cells [41, 42, 46]. 

**Figure 4:**
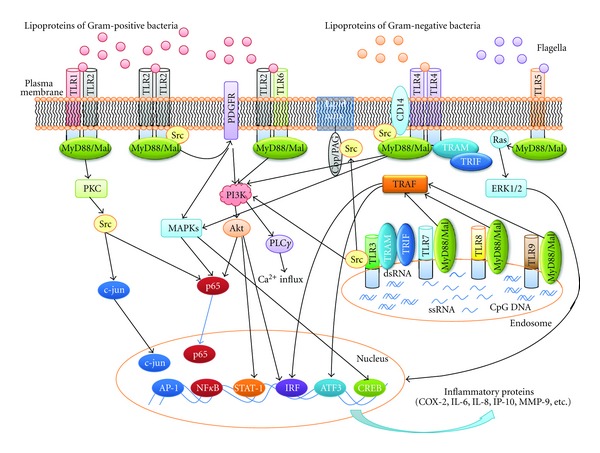
The Src-mediated signaling pathways in TLRs. In the TLR family signaling pathways, Src generally regulates the early steps of the signaling cascades.

Src is involved in the signaling pathway of all TLR molecules (Figure 4). TLR2, which is involved in various inflammatory responses, forms a homodimer or builds a complex with TLR1 or TLR6. Normally, TLR2 signaling is stimulated by lipoproteins from Gram-positive bacteria, and Src is widely utilized in TLR2 signaling cascades. In human synovial fibroblasts, TLR2 induced by lipoteichoic acid (LTA), a TLR2 ligand, phosphorylates PCK*δ*, followed by the induction of phosphorylation of Src. Consequently, activated Src increases the nuclear translocation of c-jun and p65, which requires AP-1 and NF-*κ*B and results in the induction of IL-6 [47]. It is known that LTA increases the secretion of metalloproteinase-9 (MMP-9), stimulated by a TLR2 signal in astrocytes. Hsieh et al. reported that the stimulation of TLR2 by LTA activates the Src-dependent activation of PDGFR, and this stimulation ultimately increases the cell motility caused by the induction of MMP-9 production through the activation of NF-*κ*B via PI3 K/Akt and MAPKs [48]. In airway cells, the TLR2-dependent Ca^2+^ influx mechanism is induced by the phosphorylation of tyr-616 and -761 in the cytoplasmic tail of TLR2, and these results were confirmed in *Staphylococcus aureus*-treated cells early in infection. Following the publication of this report, it was determined that PI3 K is activated by phosphorylation of TLR2 through Src, and consequently, Ca^2+^ influx is increased by activated PLC*γ* [49]. Several studies have shown that the TLR2 phosphorylation stimulated by *Helicobacter pylori* and the activation of MUC-2 and IL-8 expression induced by *Pseudomonas aeruginosa* ligands result in the activation of COX-2 in a process that includes Src family kinases [50, 51]. Recently, by inhibiting its functions using c-Src siRNA, a specific role for c-Src, and not the other Src family kinases, was described in the TLR2-dependent signaling pathway. Recently, Chun and Prince suggested that c-Src may be a TLR2-dependent kinase, demonstrated by the interaction between TLR2 and c-Src through coimmunoprecipitation studies in bacterial stimulated airway cells [52]. 

TLR3 binds to virus-related ligands, such as dsRNA and poly I:C, and it is a MyD88-independent TLR family member. TLR3 is an endosomal receptor, which is dependent on both TRIF and TRAM. Interestingly, Src is known to be independent of TRIF, and a number of studies have reported that Src interacts with the cytoplasmic tail of TLR3. Src induces antiviral events through the regulation of IRF3 and STAT-1 activities via the phosphorylation of PI3K/Akt early in infection. Surprisingly, the adaptor proteins TRIF and MyD88 are not required for this process. The effects of the new pathway are mediated by c-Src, which binds to TLR3 in dsRNA-stimulated cells. The first initiation step is mediated by dsRNA-induced phosphorylation and the activation of Src, whereas the second step results from the localization of activated Src in lipid rafts; consequently, the cytoplasmic pool of active Src is reduced. As expected, two functions for Src, its effect on cell adhesion and cell proliferation, are also inhibited by dsRNA treatment [53]. 

Unlike other TLR family members, TLR4 has a large number of adaptor molecules. Like other TLRs, TLR4 also requires several ligands for its functions. Lipopolysaccharide (LPS), a component of Gram-negative bacteria, is one of the main ligands of TLR4. Similar to TLR2, TLR4 is critical for defense mechanisms and the inflammatory responses to bacteria. It is unclear whether Src directly binds to TLR4; however, CD14, which is known to form a complex with TLR4, associates with Src, implying that Src interacts with TLR4. The activation of Src in LPS-treated macrophages is dependent on TLR4 and MyD88, and their attenuation reduces LPS-promoted phagocytosis. Phosphorylated Src is related not only to the PI3K/Akt-NF-*κ*B pathway but also to AP-1 and CREB translocation by MAPKs. Src induces a number of TLR4-dependent signals early in the inflammatory response. In addition, Src regulates LPS-induced actin cytoskeleton rearrangement and consequently adjusts morphological changes and phagocytosis in macrophages [54]. 

When macrophages are exposed to flagella, the expression of TLR5 is induced by IL-8 produced through the Src, Ras, and ERK1/2 pathways. IL-8 levels produced in response to flagella are decreased by Src-specific inhibitors and by a dominant negative Src mutant [51]. 

TLR7 and TLR8 are the TLR family members stimulated primarily by viral infection. Src blocks IP-10 production by controlling ATF3 via MyD88 and TRAF in TLR7/8 signaling, a signaling pattern that is similar to TLR3 and TLR4. Src downregulates IP-10 expression following PP2 treatment; in contrast, in Src-deficient cells, PP2 induces the production of IL-6 [55]. 

TLR9 is activated by the CpG oligodeoxynucleotide (ODN) ligand and demonstrates a signaling pattern similar to TLR7 and 8. TLR9 induces TRAF3-dependent Src activation, and this response controls IRF-3 and IRF-5 [55].

In the signaling pathways of the TLR family members, Src generally regulates the early steps of the signaling cascade. Src localizes to the membrane and rapidly interacts with receptor proteins, including TLRs. Src is critical for TLR-mediated inflammatory responses. 

### 3.2. ROS Production through NADPH Oxidase and HO-1 Defense Mechanism

**Figure 5:**
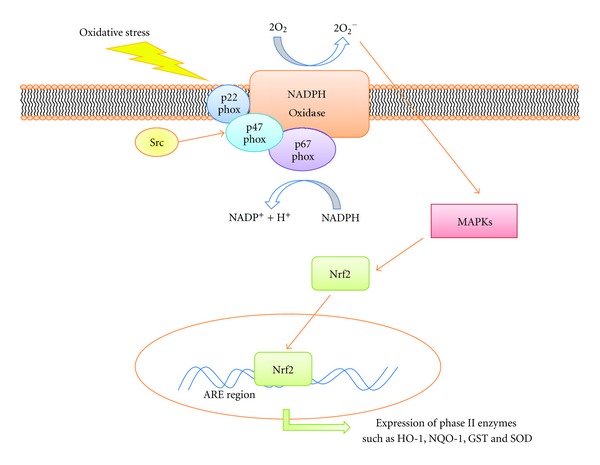
The role of Src under conditions of oxidative stress. Src has been shown to control NADPH oxidase activation and ROS production. NADPH oxidase is an enzymatic component of the production of ROS under various pathologic conditions. Activated NADPH oxidase is a multimeric protein consisting of at least three cytosolic subunits: p47phox, p67phox, and p40phox. The p47phox subunit plays a significant role in the acute activation of NADPH oxidase; the phosphorylation of p47phox is thought to inhibit intracellular interactions and promote the binding of p47phox to p22phox, thereby inducing the activation of NADPH oxidase. The expression of phase II and antioxidant enzymes is a defense mechanism that protects tissues from injury by ROS production. The phase II enzymes include the heme oxygenase-1 (HO-1), NADPH quinine oxidoreductase (NQO1), glutathione S-transferase (GST), and superoxide dismutase (SOD). These enzymes are expressed following NRF2 binding to the antioxidant response element (ARE).

Reactive oxygen species (ROS) are considered not only the main mediator of pathological tissue injury but also the cellular second messengers for a variety of cellular receptor signal transduction pathways. ROS, involving superoxide anion and hydrogen peroxide, contribute to proliferation, apoptosis, differentiation, and migration. A number of intracellular mediators, including cyclooxygenases, cytochrome P450, lipoxygenases, mitochondrial respiration, xanthine oxidase, and NADPH oxidases, regulate the enzymatic production of ROS [56–61]. In addition, Src family kinases control NADPH oxidase activation and ROS production (Figure 5). NADPH oxidase is an enzymatic component involved in the production of ROS under various pathologic conditions. Activated NADPH oxidase is a multimeric protein consisting of at least three cytosolic subunits: p47phox, p67phox, and p40phox. The p47phox subunit plays a significant role in the acute activation of NADPH oxidase; the phosphorylation of p47phox is thought to inhibit intracellular interactions and to promote the binding of p47phox to p22phox, thereby inducing the activation of NADPH oxidase [62–65]. 

Oxidative stress activates various redox-sensitive signaling pathways, including several MAPK cascades. MAPKs play roles in the production of proinflammatory cytokines, chemokines, and matrix metalloproteinases [62, 66–68]. The expression of phase II and antioxidant enzymes is a defense mechanism that protects tissue from injury by ROS production. The phase II enzymes include heme oxygenase-1 (HO-1), NADPH quinine oxidoreductase (NQO1), glutathione S-transferase (GST), and superoxide dismutase (SOD). These enzymes are expressed following the NF-E2-related factor 2 (NRF2) binding to an antioxidant response element (ARE). In particular, HO-1 is a rate-limiting enzyme for the oxidative degradation of heme to biliverdin, free iron, and carbon monoxide (CO). The protein is induced by a variety of stimuli involved under conditions of cellular stress, including cytokines, reactive oxygen species (ROS), heat shock, hypoxia, and hyperoxia [62, 69]. 

Occasionally, TLR2 and TLR4 signaling cascades induce the expression of HO-1 via one of the MAPK signaling pathways, such as p38, ERK, JNK, PI3K/Akt, and JAK-STAT. Src is a major regulator of both ROS production and cellular homeostasis, including HO-1 expression. 

## 4. The Role of Src in Tissue-Specific Macrophages and Inflammatory Diseases 

### 4.1. Alveolar Macrophages in Pulmonary Alveoli of the Lung

Alveolar macrophages (AMs) have been indicated in the recruitment of polymorphonuclear leukocytes (PMN) to the lungs during sepsis, and AMs, accompanied by inflammatory mediators, are the only resident cells required to satisfy this function. Indeed, AMs have been proposed as the critical effector cells responsible for PMN recruitment and vascular protein leakage in both acute lung injury (ALI) and acute respiratory distress syndrome (ARDS). The pharmacologic/small interfering RNA inhibition of Src decreases AM-induced endothelial NADPH oxidase activation and PMN migration [64, 70–74]. The levels of proinflammatory mediators, such as tumor necrosis factor (TNF)-*α*, interleukin (IL)-1*β*, and macrophage inflammatory protein (MIP)-2, in bronchoalveolar lavage fluids and the expression of TNF-*α* and IL-1*β* mRNA in lung tissue are increased by exposure to ultrafine TiO(2). Ultrafine TiO_2_ exposure results in the activation of important inflammatory signaling molecules, such as c-Src and p38 MAP kinase, linked to the NF-*κ*B pathway in alveolar macrophages of pulmonary tissues [75]. 

Both AMs and the lungs from *Pneumocystis murina*-infected Src triple knockout (TKO) mice express significantly higher levels of M2a macrophage markers, including RELM-*α*, Arg1, and M2a macrophage-mediated chemokines (such as CCL17 and CCL22) than wild-type mice. Wild-type and Src TKO mice do not differ in the production of IL-4 and IL-13, the primary cytokines that induce M2a macrophage polarization. *Pneumocystis murina* infection in Src TKO mice results in the elevated release of the novel IL-1 family cytokine and IL-33 in the lungs. The immunohistochemical analysis of IL-33 in the lung tissue demonstrates that it is localized mainly in the nucleus of alveolar epithelial cells [112]. 

Using the specific Src inhibitor PP1, a number of investigators have reported roles for Src in key pulmonary responses, NF-*κ*B activation, and integrin signaling for acute lung injury in mice treated with LPS. LPS treatment results in c-Src phosphorylation in the lung tissue, and phospho-c-Src is localized principally to gathered neutrophils and alveolar macrophages. PP1 inhibits the LPS-induced enhancement of total proteins in the bronchoalveolar lavage fluid and the production or activity of TNF-*α* and matrix metalloproteinase-9 as well as neutrophil recruitment. PP1 also interrupts LPS-induced NF-*κ*B activation and I*κ*B-*α* degradation. The inhibition of NF-*κ*B activation by PP1 results in a decrease in LPS-induced integrin signaling, not only by increasing the phosphorylation of integrin *β*_3_ and the focal adhesion kinase (FAK) family members, such as FAK and Pyk2, in lung tissue but also by decreasing the fibrinogen-binding activity of alveolar macrophages. Furthermore, treatment with anti-*α*_v_, anti-*β*_3_, or Arg-Gly-Asp-Ser (RGDS) inhibits the LPS-induced NF-*κ*B activation. Taken together, Src plays critical roles in the LPS-induced activation of NF-*κ*B and integrin (*α*_v_ *β*_3_) signaling during acute lung injury, which implies that Src inhibition may provide a potential treatment to ameliorate inflammatory cascades in lung injury [113]. 

### 4.2. Kupffer Cells in Liver

The liver functions in both host defense and tissue protection through hepatic cell-cell cross-talk that regulates coagulation and is essential for the inflammatory responses. When these events are not controlled correctly, secondary hepatic dysfunction may occur. Kupffer cells (KCs) are the largest population of resident macrophages in the liver. Activated KCs may cause damage to hepatocytes by the secretion of inflammatory cytokines, such as tumor necrosis factor-*α* (TNF-*α*), or by neutrophil infiltration [114–119].

Located directly in the bloodstream in the narrow liver sinusoids, KCs may under some circumstances, release vast amounts of pro-inflammatory mediators into the circulation. When the inflammatory actions of KCs are not turned off by anti-inflammatory equivalents, this may lead to systemic inflammation, such as sepsis, and damage to several additional organs, such as the kidney [114, 120, 121]. KCs have been implicated as major producers of circulating anti-inflammatory cytokines, including interleukin-6 (IL-6) and IL-10, against peritonitis and trauma. In addition, it has been reported that the production and signaling of IL-10 protects against liver injury in mice. Recently, it was reported that early increases in IL-6 and IL-10 occur in the plasma during liver surgery, whereas the levels of pro-inflammatory cytokines, such as TNF-*α* remain low. In bacterial infections, however, KCs release large amounts of TNF-*α* [119, 122–127].

Recently, the involvement of Src in TNF-*α* production has been reported in the murine macrophage cell line RAW264.7 and in J774 cells. In several studies, Src inhibitors have been shown to have no effect on IL-6 production and only limited effects on IL-10 levels, implying that the slight Src-dependent inhibition of IL-10 may be a secondary event [114, 128]. 

p38 plays an important role in IL-6 production in KCs in response to hypoxia. In contrast, the production of macrophage chemotactic protein-1 (MCP-1) is independent of p38. Other studies suggest that ERK1/2 is more important for controlling IL-6 production in KCs than p38. The disparity between the studies is likely a result of the different animal models and different stimuli used but may also reflect differential regulatory pathways in KCs following various types of injury. In male mice under hypoxic conditions, Src is activated in KCs, suggesting that Src activity is induced by hypoxia [129]. The treatment of animals with the Src inhibitor PP1 blocks the increase in Src phosphorylation as well as the subsequent elevation in IL-6 production by KCs following hypoxia. Therefore, it is likely that the Src tyrosine kinase plays a role in regulating the responses of KCs to hypoxia. [114, 130]. In summary, the activation of Src following hypoxia may result in liver disease mediated by KCs, and Src may be an inhibitory target for liver inflammation.

### 4.3. Microglia in Neural Tissue

Microglia are the resident macrophages in the brain and are generally the first cells to respond to brain injury or infection; the cells produce a graded response, including changes in morphology, increased motility, the production of inflammatory cytokines, proteases, and reactive oxygen mediators, and phagocytosis. However, the chronic or uncontrolled stimulation of microglia promotes inflammatory responses that may lead to several neurological disorders, such as Alzheimer's disease, Jacob disease, AIDS dementia, and multiple sclerosis. *In vitro*, microglia are activated by a variety of agents; none is more potent than LPS. The activation of an unidentified tyrosine kinase is an early event upstream of LPS in most cell types, and Src family kinases have been implicated in LPS signaling; however, this proposal remains controversial. Although the general tyrosine kinase inhibitor herbimycin A completely inhibits LPS-induced MARCKS, MRP, and iNOS in BV-2 cells, little evidence for the involvement of Src-like kinases has been obtained using specific Src inhibitors, such as PP1 and PP2. The unexpected increases in MARCKS and MRP have been observed at lower doses using the Src inhibitor PP1 [131–135]. 

In other neurodegenerative pathways, CD40 may be a positive regulator of Src; this was investigated in microglia challenged with CD40L. The CD40L-mediated TNF-*α* production in microglia is dependent on p44/42 MAPK, and Src activation might bridge the stimulation of microglial CD40 and the consequent activation of p44/42 MAPK. Therefore, microglia cotreated with CD40L and the Src family kinase inhibitor PP1 demonstrate a marked reduction in both p44/42 MAPK activation and TNF-*α* secretion by these cells. The activation of Src is required for the transduction of p44/42 MAPK-dependent TNF-*α* production following CD40 ligation. This is especially interesting when considered in conjunction with the stimulation of microglial CD45, in which the cotreatment with CD40L and anti-CD45 mAb results in the dramatic inhibition of Src and downstream p44/42 MAPK activities as well as TNF-*α* secretion [136, 137]. Therefore, Src might be a suitable therapeutic target for the treatment of neurodegenerative diseases involving the activation of pathological microglia.

### 4.4. Osteoclasts in Bone

Osteoclasts are multinucleated, terminally differentiated cells that degrade the mineralized matrix during normal and pathological bone turnover. Osteoclastic bone resorption includes the proliferation of the hemopoietic osteoclast progenitors to bone, their differentiation and fusion to form multinucleated cells, and the migration of osteoclasts to the resorptive bone. Many researchers have suggested that the activation of Src is associated with the movement from stable focal adhesions with actin fibers to a more dynamic podosome complex, possibly by regulating cell motility [138]. Although several studies have proposed a role for Src activity in the spreading and migration of cells, it remains unclear whether the catalytic activation of Src is required for general osteoclast functions. It has been reported previously that the osteoclast-specific expression of kinase-dead Src mutants rescues the Src−/− osteopetrotic phenotype [139], suggesting that c-Src may play a role as an adaptor molecule and that c-Src activity may not be important in bone resorption. On the other hand, recent reports have demonstrated that the down- or upregulation of c-Src activity regulates osteoclastic bone resorption not only* in vitro* but also *in vivo*, leaving the issue of the contribution of c-Src activity unresolved [140–146].

c-Src is required for retaining the cyclooxygenase (COX) activity in osteoclasts. Furthermore, COX activity is required for the bone-resorbing activity of mature osteoclasts. The most notable defect in SYF cells is the decrease in cell proliferation and motility, which are both ATP-dependent events. The c-Src in mitochondria modulates COX activity, and c-Src/COX signaling is important for the bone-resorbing activity of osteoclasts. A reduction in the COX activity mediated by c-Src may be a characteristic of the osteopetrotic phenotype of c-Src−/− mice [140].

Recent studies have indicated that osteoclasts are involved in the pathogenesis of bone and joint destruction and are a potentially potent therapeutic target for the treatment of rheumatoid arthritis (RA). Therapies that diminish osteoclast formation or function may improve the progression of bone degradation. A cure for RA is unlikely until its etiology is explained; however, the suppression of osteoclast activity by modulating various signaling pathways, including c-Src, will likely be pursued as a novel therapeutic approach for preventing the joint breakdown associated with RA [140].

## 5. Therapeutic Approaches Using Src Inhibitors

Understanding the functional and biological significance of Src in tumorigenesis has led to the development of novel Src inhibitors for therapeutic purposes. In recent years, increasing numbers of chemical compounds have been designed and synthesized as novel inhibitors of Src. Understanding the structure of Src has allowed the development of selective and strong inhibitors that are structurally optimized to bind target areas, such as the ATP-binding motif or other allosteric sites. Bosutinib (SKI-606), dasatinib (BMS-354825), saracatinib (AZD0530), KX2-391, and NVP-BHG712 are examples of recently developed Src inhibitors, exhibiting IC_50_ values ranging from 0.001 to 0.3 *μ*M. In addition to the direct assessment of enzymatic activity, the biological activities of these inhibitors have been tested primarily in cancer disease settings; for example, studies have addressed the ability of Src inhibitors to reduce the progression of breast, colon, and thyroid cancers [147]. Because trials have not assessed the immunopharmacological efficacy of these inhibitors under inflammatory conditions either *in vitro *or *in vivo*, systematic approaches could lead to the discovery of promising immunosuppressive or anti-inflammatory drugs that target Src during inflammatory responses [148–152].

### 5.1. Plant Extracts

Although few experimental trials of selective Src inhibitors have been reported, evidence suggests that plant extracts act by suppressing the Src-related pathways (Table 4). For example, *Sorbus commixta *water extract attenuates TLR4/MyD88-mediated NF-*κ*B translocation by inhibiting Src and Syk in murine macrophages [101]. It has also been reported that treatment with an ethanol extract of *Phaseolus angularis *beans results in a dose-dependent reduction in the production of NO and PGE_2_ in LPS-, poly(I:C-), and pam3CSK-activated RAW264.7 cells through a transcriptional mechanism [100]. *Polygonum hydropiper *L.extract has been shown to regulate the activation of NF-*κ*B, AP-1, and CREB by effectively inhibiting upstream inflammatory signals, including Syk, Src, and IRAK1 [98]. Notably, treatment with an ethanol extract of *Sanguisorba officinalis *decreased the production of inflammatory mediators in LPS-activated RAW264.7 cells and peritoneal macrophages by suppressing the activity of IKK/I*κ*B/NF-*κ*B, Akt, ERK1/2, and JNK. Moreover, this treatment inhibited the phosphorylation and kinase activity of Src [102]. The plant extracts that inhibit Src are summarized in Table 4. 

### 5.2. Natural Products

In recent studies, several compounds from natural products have been reported to inhibit Src activity and inflammatory responses (Table 5). Kahweol and arctigenin decreased the protein levels of nuclear factor of activated T-cell cytoplasmic-1 (NFATc1), a major regulator of osteoclast differentiation, and downregulated the osteoclast markers transcriptionally modulated by NFATc1, such as Src and cathepsin K [110, 111]. The inhibitory effects of glabridin, a flavonoid purified from licorice root, in murine osteoclast progenitor RAW264.7 cells are also mediated by the RANKL-induced expression of signaling molecules (TRAF6, GAB2, ERK2, JNK1, and MKK7) and osteoclast survival-related signaling pathways involved in c-Src, PI3 K, and Akt2 [108]. 

Cytochalasin B, which blocks actin polymerization, decreases both the LPS-induced phosphorylation and kinase activity of Src without changing the total protein levels, implying that Src is a potential pharmacological target of actin cytoskeleton rearrangement. Furthermore, the direct association of Src with actin was confirmed by immunoprecipitation analysis performed using a GFP-actin wild-type and HA-tagged Src [104]. Therefore, actin cytoskeleton rearrangements may be a key event in the regulation of inflammatory responses that control the activity of Src and its downstream signaling proteins [104]. Moreover, morelloflavone, a biflavonoid, has been shown to block the migration of vascular smooth muscle cells through the inhibition of multiple migration-related kinases, such as focal adhesion kinase, c-Src, ERK, and RhoA [109].

Several newly synthesized derivatives of kojic acid, a compound with known antiinflammatory, anti-proliferative, and antioxidative properties, modulated glioma cell proliferation and TLR4-mediated activation in macrophage-managed tumor microenvironments [106]. The anti-inflammatory activities of kojic acid derivatives were evaluated by determining the production of nitric oxide (NO) and cytokines in macrophages (RAW264.7 cells) stimulated with LPS. Among the various derivatives tested, RHS-0110 exhibited the strongest inhibitory activity on the Src phosphorylation levels. Lower or noncytotoxic doses of kojic acid derivatives also downregulated LPS-induced NO production and interleukin- (IL-)6 expression in RAW264.7 cells [106]. We suggest that natural products that inhibit Src activity and inflammatory responses exhibit strong immunosuppressive and anti-inflammatory properties; therefore, they are potential candidates for anti-inflammatory therapeutic drugs.

### 5.3. Redox-Sensitive Cysteine Residues

Oxidative stress has been implicated in the progression of many inflammatory diseases, including pulmonary disease, gastritis, neurodegenerative disorders, atherosclerosis, and bowel disease [153–157]. Many reports have suggested that cysteine is an important target of redox-mediated signaling and inflammatory therapy [158].

In addition to the phosphorylation-based regulation of Src, recent studies have indicated a possible role for cysteine modification in the regulation of the kinase. Of the ten cysteine residues scattered throughout the Src protein, three (Cys-185, Cys-238, and Cys-245) are located in the SH2 domain, two (Cys-277 and Cys-400) are in the N-terminal portion of the catalytic domain, four (Cys-483, Cys-487, Cys-496, and Cys-498) are conjugated to a cluster at the bottom of the catalytic domain, and one (Cys-520) is in the C-terminal end of the protein. Of these cysteines, two in the SH2 domain (Cys-238 and Cys-245) and three in kinase domain (Cys-400, Cys-487, and Cys-498) are highly conserved among the Src family kinases [159]. Specially, four cysteines in the C terminus of the c-Src catalytic domain, including Cys-483, Cys-487, Cys-496, and Cys-498, comprise the cysteine-clustered motif (CC motif) [159, 160].

The substitution of these cysteine residues renders Src refractory to inactivation by SH-alkylating agents and mercuric ions, such as HgCl_2_, which have high affinity for the thiols in cysteines [160]. Interestingly, hypoxia-induced oxidative stress causes the differential redox regulation of Src. It is known that Cys-245 and Cys-487 are involved in the oxidation/activation of Src during hypoxia; however, studies using mutants in either Cys-245 or Cys-487 demonstrated that the oxidation of Cys-487 is critical for increasing the kinase activity of Src, indicating the formation of intermolecular Src S-S adducts [161, 162]. Kemble and Sun also suggested a mechanism for the direct oxidative inactivation of Src specifically. Occasionally, this inactivation of Src results in the oxidation of a specific cysteine residue (Cys-277), which in turn forms a Src homodimer via a disulfide bridge at Cys-277, located in the Gly loop in the catalytic domain of Src [163]. Studies to prove the anti-inflammatory functions of these novel Src inhibitors that target cysteine residues are actively ongoing.

## 6. Summary

Numerous studies have revealed that Src plays pivotal roles in macrophage-mediated inflammatory responses. Importantly, a variety of inflammatory diseases is closely related to macrophage activation. The critical roles of Src in macrophage activation prompted us to consider that the inhibition of Src activity may be a useful therapeutic strategy for macrophage-mediated diseases. Recently, several studies have investigated the possibility that Src inhibitors are useful for this purpose. Considering that the Src CC motif is a potent target for anti-inflammatory activities, future studies may develop CC motif-targeted compounds for Src-targeted immunomodulatory drugs. We expect that novel and safe Src inhibitors exhibiting strong immunosuppressive and anti-inflammatory properties will contribute to the development of innovative therapies for the treatment of macrophage-mediated diseases.

## Figures and Tables

**Table 1:** Src family kinases and their functions in immune responses.

Molecule	Distribution	Specific functions in immune responses	Reference
Src	Ubiquitous	- See Sections 2, 3, and 4	
Fyn	Ubiquitous	- CD5 glycoprotein-mediated T-cell inhibition by inhibitory phosphorylation of Fyn	[76]
		- Rac and stress kinase activation in TCR signaling by Fyn	
Yes	Ubiquitous	- LTB4-mediated degranulation of human neutrophils by Yes activation	[77]
Frk	Ubiquitous	- Unclear	
Blk	G, Mo, Ma, B	- Preferentially expression in B-cell lineage. Control of proliferation during B-cell development	[78]
Fgr	G, Mo, Ma	- Inhibition of Hck and Fgr kinase activity	[79]
		- Inhibition of beta 2 integrin receptor and Syk kinase signaling by Fgr	[80]
Hck	G, Mo, Ma	- Inhibition of Hck and Fgr kinase activity	[79]
Lck	T, B	- Modulation of signaling and cellular fate of B-1 cells	[81]
		- Promoting B-cell development	[82]
Lyn	P, G, Mo, Ma, B	- Redox sensor mediating initial neutrophil recruitment to wounds	[83]
		- Inhibition of platelet aggregation with PECAM-1	[84]

^∗^Data from http://www.proteinkinase.de. G: granulocyte, Mo: monocyte, Ma: macrophage, B: B cell, T: T cell, and P: platelet.

**Table 2:** Src-interacting molecules corresponding to SH2 or SH3.

Domain	Interacting molecules	Reference
	SHP-1 protein tyrosine phosphatase	[85]
	Protein tyrosine phosphatase-1B	[86]
	Nonreceptor type 1	[87]
SH2 (149–239)	Dual-adaptor for phosphotyrosine and 3-phosphoinositides-1	[88]
	Heterogeneous nuclear ribonucleoprotein K-1	[89]
	CRK-associated substrate	[90]
	Disabled-1	[91]

	Cyclin-dependent kinase-5	[92]
	KCNB1	[93]
	p21-activated kinase-2	[94]
SH3 (87–144)	CRK-associated substrate	[90]
	Vinculin	[95]
	Fragile histidine triad protein	[96]
	GRB2	[87]

**Table 3:** The classification of TLR family.

Receptor	Ligand	Adapter molecules	Location	Cell type
TLR1 + TLR2	Bacterial lipoproteins	MyD88/Mal	Plasma membrane	Monocytes/macrophages A subset of dendritic cells B lymphocytes
TLR2 + ?	GPI anchors (parasites), bacterial porins, and HMGB1	MyD88/Mal	Plasma membrane	Monocytes/macrophages A subset of dendritic cells B lymphocytes
TLR3	dsRNA, poly I:C	TRIF	Intracellular membrane	Dendritic cells B lymphocytes
TLR4	LPS, HSPs, HMGB1, some viral proteins	MyD88/Mal/TRAM/TRIF	Plasma membrane	Monocytes/macrophages Myeloid dendritic cells Mast cells B lymphocytes Intestinal epithelium
TLR5	Bacterial flagellin	MyD88	Plasma membrane	Monocyte/macrophages A subset of dendritic cells Intestinal epithelium
TLR2 + TLR6	Bacterial lipoproteins from mycoplasma, lipoteichoic acid, and yeast cell wall mannans	MyD88/Mal	Plasma membrane	Monocytes/macrophages Mast cells B lymphocytes
TLR7	Imidazoquinoline ssRNA (viral)	MyD88	Intracellular membrane	Monocytes/macrophages Plasmacytoid dendritic cells B lymphocytes
TLR8	Imidazoquinoline ssRNA (viral)	MyD88	Intracellular membrane	Monocytes/macrophages A subset of dendritic cells Mast cells
TLR9	CpG-containing DNA (viral and bacterial)	MyD88	Intracellular membrane	Monocytes/macrophages A subset of dendritic cells Mast cells B cell
TLR10	Unknown	MyD88	Plasma membrane	B lymphocytes Dendritic cells Eosinophils
TLR11 (only mouse and rat)	Toxoplasma profilin	MyD88	Plasma membrane	Monocytes/macrophages A subset of dendritic cells Mast cells
TLR12 (only mouse and rat)	Unknown	MyD88	Plasma membrane	Neurons
TLR13 (only mouse and rat)	Unknown	MyD88, TAK-1	Plasma membrane	

**Table 4:** Plant extracts inhibiting Src activation in macrophages.

Plant	Target Src pathway	Reference
*Archidendron clypearia *	Src/NF-*κ*B-targeted inhibition of LPS-induced macrophage activation and dextran sodium sulfate-induced colitis	[97]
*Polygonum hydropiper *	Suppression of Src/Syk/NF-*κ*B and IRAK/AP-1/CREB pathways and dextran sodium sulfate-induced colitis	[98]
*Cinnamomum cassia *	Suppression of Src/Syk-mediated NF-*κ*B activation and antigastritis	[99]
*Phaseolus angularis *beans	NO and PGE_2_ production mediated by the suppression of NF-*κ*B and AP-1 activation signaling cascade and antigastritis	[100]
*Sorbus commixta *	Suppression of the inflammatory signaling cascade composed of Src, Syk, and NF-*κ*B.	[101]
*Sanguisorba officinalis *	NO and PGE_2_ production mediated by the suppression of NF-*κ*B and AP-1 activation signaling cascade	[102]

**Table 5:** Naturally occurring compounds inhibiting Src pathway activation in macrophages.

Compound	Action target of Src	Reference
Saurolactam	Inhibition of osteoclast differentiation and stimulation of apoptosis in mature osteoclasts.	[103]
Cytochalasin B	Suppression of actin cytoskeleton rearrangement	[104]
Butyrate	Reduction of lipopolysaccharide-mediated macrophage migration	[105]
RHS-0110 (Kojic acid derivative)	Suppression of LPS-induced NO production and interleukin- (IL-) 6 expression	[106]
Maslinic acid	Suppression of RANKL-induced osteoclastogenesis	[107]
Glabridin	Suppression of RANKL-induced osteoclastogenesis	[108]
Morelloflavone	Inhibition of migration-related kinases, amelioration of atherosclerosis in mice	[109]
Arctigenin	Suppression of (RANKL-) mediated osteoclast differentiation	[110]
Kahweol	Prevention of osteoclastogenesis	[111]
